# The association between melatonin and suicide: a nationwide cohort study

**DOI:** 10.1192/j.eurpsy.2022.2185

**Published:** 2022-09-01

**Authors:** N. Høier, T. Madsen, A. Spira, K. Hawton, P. Jennum, M. Nordentoft, A. Erlangsen

**Affiliations:** 1 Mental Health Centre Copenhagen (CORE), Danish Research Institute For Suicide Prevention, Hellerup, Denmark; 2 Mental Health Center Copenhagen, Core-copenhagen Research Center For Mental Health, Hellerup, Denmark; 3 Johns Hopkins Bloomberg School of Public Health, Department Of Mental Health, Baltimore, United States of America; 4 University of Oxford, Centre For Suicide Research, Oxford, United Kingdom; 5 Danish Center for Sleep Medicine, Rigshospitalet, Copenhagen University, Copenhagen N, Denmark; 6 CORE-Copenhagen Research Center for Mental Health, Mental Health Center Copenhagen, Hellerup, Denmark

**Keywords:** sleep medicine, melatonin, Pharmacology, Suicide

## Abstract

**Introduction:**

Melatonin is often prescribed to patients experiencing sleep disturbances, which has been linked to elevated risks of suicide. However, it remains to be assessed whether melatonin is associated with suicide and suicide attempts.

**Objectives:**

We aimed to investigate whether individuals in treatment with melatonin had higher rates of suicide and suicide attempt when compared to individuals not in treatment.

**Methods:**

Using longitudinal data on all persons aged 10+ years living in Denmark between 2007-2016 were obtained. Data from the National Prescription Register was used to identify periods of being in treatment with melatonin based on number of tablets and daily defined dose. Suicide and suicide attempt were identified in hospital and cause of death registries.

**Results:**

Among 5,798,923 included individuals, 10,577 (0.18%) were in treatment with melatonin (mean treatment length 50 days). Out of 5,952 individuals who died by suicide, 22 (0.37%) were in melatonin treatment, while 134 (0.53%) out of 25,136 had a first suicide attempt. After adjustment for sex and age-group, people in treatment with melatonin were found to have a higher rate of suicide (IRR: 4.2; 95% CI, 2.7-6.4) and suicide attempt (IRR: 6.7-fold (95% CI, 5.7-7.9) when compared to those not in treatment.

**Conclusions:**

Treatment with melatonin was associated with higher rates of suicide and suicide attempt. The association might be explained through mediators, such as psychiatric comorbidity and sleep disorders. Our findings indicate that attention towards these issues might be warranted.

**Disclosure:**

No significant relationships.

